# The natural history of, and risk factors for, progressive Chronic Kidney Disease (CKD): the Renal Impairment in Secondary care (RIISC) study; rationale and protocol

**DOI:** 10.1186/1471-2369-14-95

**Published:** 2013-04-25

**Authors:** Stephanie Stringer, Praveen Sharma, Mary Dutton, Mark Jesky, Khai Ng, Okdeep Kaur, Iain Chapple, Thomas Dietrich, Charles Ferro, Paul Cockwell

**Affiliations:** 1 Department of Nephrology, University Hospital Birmingham, Birmingham B15 2WB, UK; 2 School of Immunity and Infection, University of Birmingham, Birmingham B15 2TT, UK; 3 Periodontal Research Group, School of Dentistry, University of Birmingham, Birmingham B4 6NN, UK; 4 MRC Centre for Immune Regulation, Birmingham, UK

**Keywords:** CKD progression, Observational cohort study, Inflammation, Arterial stiffness, Periodontitis

## Abstract

**Background:**

Chronic kidney disease (CKD) affects up to 16% of the adult population and is associated with significant morbidity and mortality. People at highest risk from progressive CKD are defined by a sustained decline in estimated glomerular filtration rate (eGFR) and/or the presence of significant albuminuria/proteinuria and/or more advanced CKD. Accurate mapping of the bio-clinical determinants of this group will enable improved risk stratification and direct the development of better targeted management for people with CKD.

**Methods/Design:**

The Renal Impairment In Secondary Care study is a prospective, observational cohort study, patients with CKD 4 and 5 or CKD 3 and either accelerated progression and/or proteinuria who are managed in secondary care are eligible to participate. Participants undergo a detailed bio-clinical assessment that includes measures of vascular health, periodontal health, quality of life and socio-economic status, clinical assessment and collection of samples for biomarker analysis. The assessments take place at baseline, and at six, 18, 36, 60 and 120 months; the outcomes of interest include cardiovascular events, progression to end stage kidney disease and death.

**Discussion:**

The determinants of progression of chronic kidney disease are not fully understood though there are a number of proposed risk factors for progression (both traditional and novel). This study will provide a detailed bio-clinical phenotype of patients with high-risk chronic kidney disease (high risk of both progression and cardiovascular events) and will repeatedly assess them over a prolonged follow up period. Recruitment commenced in Autumn 2010 and will provide many outputs that will add to the evidence base for progressive chronic kidney disease.

## Background

Chronic kidney disease (CKD) is strongly associated with poor health outcomes [1-5]. It affects up to 16% of the adult population in the UK and internationally [6,7]. Those at highest risk from progressive CKD have an accelerated deterioration of kidney function, significant albuminuria, and/or more advanced CKD at inception [8,9]. The large majority of people with CKD die before they reach end-stage kidney disease (ESKD) and have an increased risk of cardiovascular disease (CVD) that is directly related to the severity of their kidney disease [10,11].

These associations were recently explored in meta-analyses of both general and high risk cohorts conducted by the Chronic Kidney Disease Prognosis Consortium; both low eGFR and proteinuria were independent predictors of acute kidney injury, ESKD and progression of CKD independent of other cardiovascular risk factors [7]. In the same cohorts low eGFR and albuminuria were independently associated with all cause mortality as well as cardiovascular mortality [12,13].

Despite the strong association with poor outcomes kidney disease has the lowest evidence base of any major medical specialty, including a lack of knowledge of the determinants of poor health outcomes in CKD [14]. A recent comprehensive systematic review, struggled to find large, high quality randomised controlled trials (RCTs) from which to make strong recommendations; the authors found that evidence of outcomes associated with interventions in CKD patients was sparse and often derived from *post hoc* analyses of subgroups of patients enrolled in trials. Few trials reported or systematically collected information about adverse events, suggesting the possibility of selective reporting and publication bias [15,16].

Furthermore, a significant component of enhanced cardiovascular risk in CKD is independent of traditional risk factors for CVD and premature mortality [6,17], therefore pathways that link CKD and CVD may involve novel patho-biological processes [18]. Better understanding of these pathways is essential for the development of new treatments for patients with CKD.

The studies that have reported outcomes associated with CKD on a population basis [6,7] have significants limitations in directing studies of intervention for people with CKD. Firstly, they provide limited bioclinical data beyond the measurement of kidney function by equations derived from serum creatinine and/or kidney damage as assessed by the presence of protein in the urine. Secondly, the generalisability of the findings of earlier studies to current patient populations is uncertain. For example, the widespread use of ACE inhibitors or angiotensin receptor blockers for progressive proteinuric kidney disease has changed the natural history of the disease over the last decade [19-21]. Clinical data on anaemia targets, the use of statins in patients with CKD and enhanced multidisciplinary team management of people with CKD may also be contributing to better long-term outcomes [22-25]. Furthermore, there may be no survival benefit associated with an early start on dialysis, therefore a requirement for renal replacement therapy reported as a surrogate end-point in previous studies may have limited current relevance as clinicians focus less on estimate glomerular filtration rate (eGFR) based commencement of dialysis and more on the commencement of dialysis based on symptoms of advanced CKD [26].

To address these limitations carefully designed studies that specifically address natural history are necessary, with the accurate acquisition of cohorts of patients with prospectively collected enhanced clinical datasets and incorporating the collection and storage of biological samples that allow biomarker identification and characterisation. These cohorts require careful long-term follow-up in order to address temporality of exposure and outcome variables.

The gold standard methodology for testing hypotheses is the RCT [27,28]; however with the scarcity of high quality RCTs in renal medicine [29], and with the inherent limitation of this approach to address some research questions, in particular those involving the identification of risk factors associated with certain outcomes, RCTs are not the most appropriate approach for some studies and observational cohorts can generate results that are highly important for improving clinical practice [28]; these types of studies can include participants with a greater spectrum of disease severity and co-morbidity than an RCT [27] and address a broader range of hypotheses.

To date there have been seven prospective observational cohort studies specifically designed to provide additional information about the natural history of CKD based on enhanced phenotyping and clinical follow-up. These studies and the populations of which they are comprised are listed in Table 1. The studies differ in three major ways, namely: (i) criteria for recruitment; (ii) the information collected; (iii) outcome measures employed. The primary aims of each study and the composition of bio-clinical assessments used are summarised in Table 2.

**Table 1:** Existing CKD cohort studies

**Cohort**	**Population**	**Year commenced**	**Number recruited**
Chronic Renal Impairment in Birmingham (CRIB) [30]	CKD with Creatinine >1.47 mg/dl (130 mmol/l) pre-dialysis	1997	369 (completed)
Mild to Moderate Kidney Disease study (MMKD) [31]	Patients who had attended secondary care nephrology clinics at least twice	1997	277 (completed)
Longitudinal Chronic Kidney Disease Study (LCKD) [32]	Secondary care, GFR < 50 ml/min on two occasions	2000	820 (completed)
Chronic Renal Insufficiency Standards Implementation Study (CRISIS) [33]	Secondary care stage 3–5 CKD (pre-dialysis)	2002	1325 (completed)
Chronic Renal Insufficiency Cohort (CRIC) [34,35]	Secondary care, all CKD stages	2003	3612 (still recruiting)
Study for the evaluation of early kidney disease (SEEK) [36]	Predominantly primary care (29% from secondary care), inclusion based upon single eGFR ≤60 ml/min	2004	1814 (completed)
Renal Risk In Derby (R^2^ID) [37]	Primary care, eGFR 30-59 ml/min on more than two occasions three months apart	2008	1741 (completed)

**Table 2:** The core phenotyping and primary aims of CKD observational cohort studies

**Cohort**	**Primary aims**	**Cardiovascular phenotyping**	**Other bio-clinical phenotyping**
CRIB [30,38]	To explore the relationship between CKD and CVD in individuals not receiving dialysis	12 lead ECG for assessment of LVH	Medical history; height, weight and blood pressure; urine and non-fasted blood, creatinine and eGFR (MDRD)
MMKD [31]	To explore the natural history of mild to moderate CKD and identify possible biomakers of progression		Medical history, clinical examination and blood and urine collection for biomarker analysis
LCKD [32]	To describe the course of the disease and the determinants of patient outcomes	In phase II patients from the initial phase invited to undergo echocardiography, flow mediated vasodilation, pulse wave velocity, 24 hr heart rate monitoring and spiral CT. All these investigations are done on two occasions one year apart	Health related QoL (SF36); co-morbidites and medications; blood ans urine samples for biomarker analysis
CRISIS [33]	To describe the risk factors associated with renal progression	Augmentation index and carotid-radial pulse wave velocity (SphygmoCor system); two blood pressure readings	Medical History; medication history; Blood and urine for biomarker analysis
CRIC [35,39,40]	To examine the risk factors for both the progression of CKD and the development of CVD	12 lead ECG and echocardiography at years 1 and 4 of follow up, sprial CT in a third of participants, pulse wave velocity (PWV) measured in 2564 participants	eGFR (MDRD in all, iothalamate in a sub-group), annual blood and urine sampling for biomarker analysis
SEEK [36]	To examine the prevalence of abnormalities in PTH, vitamin D, phosphate and calcium in patients with CKD	None	Medical history; medication history; blood and urine samples
R^2^ID [37]	To assess the need for specialist referral in a primary care CKD population and measure rate of change of kidney function	Pulse wave velocity and augmentation index (Vicorder system), advanced glycation end products (AGE reader system)	Socio-economic measures (IMD score); medical and medication history; anthropomorphic measures; blood and urine sampling;

*ECG*, Electrocardiogram, *LVH*, left ventricular hypertrophy, *MDRD*, modification of diet in renal disease formula, *SF36*, short form 36 for the assessment of quality of life, *QoL*, Quality of Life, *PTH*, parathyroid hormone, *AGE*, Advanced glycation end products.

To date no cohort has recruited patients with a specific focus on those at highest risk from their CKD as a consequence of rate of decline of kidney function and/or proteinuria. They have focused on recruitment based on CKD stage. This is an important distinction; whilst CKD stage itself confers an increment of risk, a large proportion of the risk is associated with the rate of change of kidney function and/or the presence of proteinuria [41]. Developing a study aimed specifically at high risk CKD patients allows the accurate bio-clinical phenotyping of a tightly defined group of patients with progressive CKD and the identification of risk factors that are associated with clinical outcomes in this group, including early mortality and progression of CKD.

One under studied co-morbidity, which impacts upon the systemic inflammatory burden is periodontitis, which is the most prevalent chronic inflammatory disease of humans [42]. The oral microbiome comprises 1200 phylotypes [43] with direct access to the gingival micro-circulation, where local inflammatory responses can persist long-term and are associated with elevated systemic inflammation and risk for CVD [44], rheumatoid arthritis [45] and adverse diabetes outcomes [46].

To address this we have established a study to identify the natural history of CKD in patients at highest risk from their renal phenotype. This study is named Renal Insufficiency In Secondary Care (RIISC); the hypotheses that drove the development of the RIISC cohort are shown in Table 3.

**Table 3:** The hypotheses that the RIISC cohort aims to address

Risk factor for CKD progression	There are unidentified novel risk factors for CKD progression
	Novel risk factors are a component of the enhanced cardiovascular risk experienced by individuals with CKD
	The presence of periodontitis contributes to both progressive CKD and CVD risk by a mechanism of increasing the systemic inflammatory burden
The phenotype of progressive CKD	The vascular, renal, oral and systemic inflammatory phenotypes of patients with progressive CKD are inter-related.
	The establishment of a cohort of high risk CKD patients with detailed vascular, renal, oral and systemic inflammatory phenotyping at various time-points over a ten year period will provide data on the changing patho-biology of progressive CKD.

Patients who meet criteria for secondary care follow-up as defined by the UK based National Institute of Clinical Excellence (NICE) CKD guidelines are eligible to participate in RIISC [47]. The main aims of RIISC are to (i) determine what factors confer high risk of progression of CKD and development and progression of CVD; (ii) enable the stratification of risk; (iii) assess the relationship between CKD, oral and systemic inflammation and vascular injury. The project will address a major shortfall in knowledge with the goal of improving clinical outcomes.

## Methods/design

The study protocol has been approved by the South Birmingham Local Research Ethics committee (reference 10/H1207/6) and University Hospitals Birmingham Research and Development department (reference RRK3917). The inclusion and exclusion criteria are shown in Table 4. Patients with progressive CKD are identified from secondary care renal clinics, where they have been under follow-up for at least one year, by an automated IT system that reports albumin creatinine ratio (ACR) data and generates an automated assessment of the rate of decline of kidney function (see below). Written information is sent to patients in advance of their attendance at the study clinic; for those patients who do not speak English, translated information is sent in audio format (as it is known that patients who do not speak English may not be able to read in their own language) [48]. Informed consent is obtained at the study index visit according to GCP guidance; patients who do not wish to participate continue to be followed up in standard nephrology clinics.

**Table 4:** Inclusion and exclusion criteria

**Inclusion criteria (at least one required)**	**Exclusion criteria**
CKD stage 3 with either decline of eGFR† of ≥5 mls/min/year or ≥10 mls/min/5years or ACR* ≥70 mg/mmol on three occasions	Renal Replacement therapy
CKD 4 or 5 (pre-dialysis)	Immunosuppression

† estimated Glomerular Filtration Rate.

* Albumin Creatinine Ratio.

**Figure 1:**
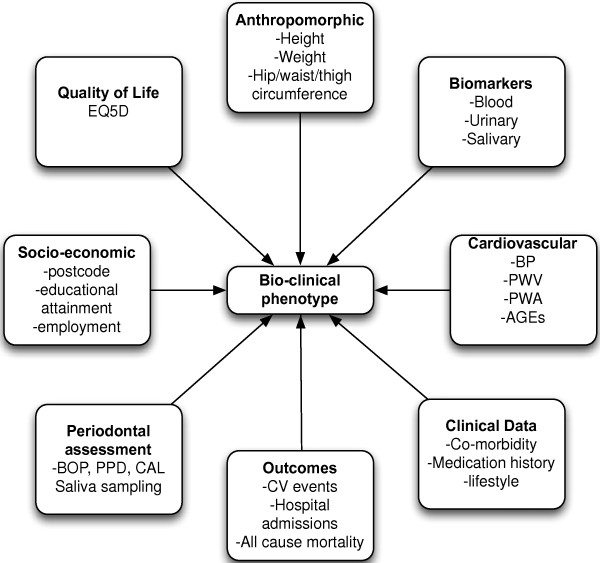
Overview of the bio-clinical assessment.

**Figure 2:**
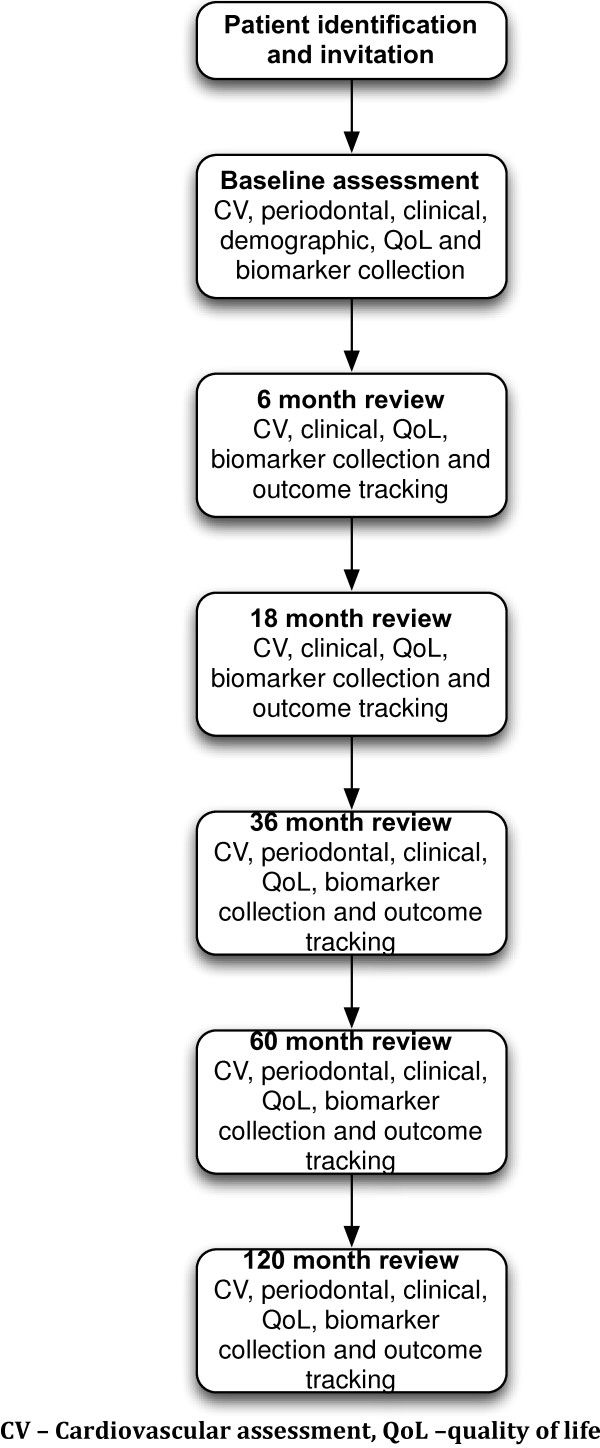
Timeline of study visits and assessments performed.

Participants undergo a detailed bio-clinical assessment an overview of which is shown in Figure 1. The study reviews are integrated into the routine clinical follow up process and participants are followed up for 10 years or until they reach a defined clinical end-point (ESKD or death). The time-points of the study visits and the data collected at each time-point is shown in Figure 2.

Each study visit is arranged around routine clinic visits, so patients are not required to attend the clinic more frequently than their clinical condition and current guidelines dictate [47]. Outcomes and endpoints embedded in RIISC are shown in Table 5. Patients who reach ESKD are withdrawn from further follow-up, although ethical permission to continue to collect cardiovascular events and mortality data on these patients has been obtained.

**Table 5:** Clinical outcomes and endpoints

**Clinical outcomes**	**Clinical endpoints**
Cardiovascular events	Death
Hospitalisations (and days hospitalised)	ESKD (as defined a requirement for renal replacement therapy)
Progression of CKD as measured by decline in eGFR calculated by linear regression	

All participants undergo a full clinical history and examination, including past history and family history of kidney disease, social history and employment history. Co-morbidity is scored using the Charlson index [49]. The study specific assessments and the evidence base for these are described below.

### Assessment of rate of renal decline

An assessment of the rate of decline of renal function is important for both the identification of potential participants and to measure the outcomes of those participants. However measuring rate of change of kidney function is complex (because renal decline is rarely a linear phenomenon) [50] and there is no gold standard methodology. A linear regression method is used to measure renal decline, utilising eGFR as calculated by the 4-variable modification of diet in renal disease (MDRD) formula with serum creatinine that is IDMS traceable. For screening rate of decline, each potential participant must have at least 6 eGFR results (obtained between a 12 and 60 month period), to allow an accurate assessment of the rate of change of eGFR with time [51].

In the MDRD study the intra-test variability of the creatinine based eGFR was 9.4%, the variability being greatest at the extremes of GFR [52]. In a study examining the accuracy of creatinine based eGFR equations in clinical studies in comparison to iothalamate based GFR measurements, Levey at al in 1993 recommended that to reduce the intra-test variability at least four measures should be used [53]. This approach has now been validated by a number of studies and there is consensus that using between four and six eGFRs collected over a period of at least one year is a more accurate way of assessing decline than percentage change in creatinine based on two results [51,54,55].

The presence of significant albuminuria is also part of the inclusion criteria, early work on urinary albumin creatinine ratio measurement found that there was significant intra-test variability associated with this method (around 60%) [56]. To reduce the impact of this variability potential participants are required to have three ACR measurements greater than 70mg/mmol (the cut off limit defined by NICE as “higher level proteinuria” [47].

### Assessment of socioeconomic status and quality of life (QoL)

Socioeconomic factors are known to influence both the prevalence and severity of chronic disease [57,58]. Population based studies conducted in both the United States and Europe have demonstrated an increased risk of CKD in individuals of lower socioeconomic status (SES) [54,59-65]. The influence of race and SES has been explored in North American studies where African-American subjects were more likely to be of lower SES and have a corresponding increased risk of prevalence and severity of CKD [37,49-54]. However the relationship between SES and CKD is complicated by the influence of other established risk factors for CKD, which are related to both CKD and reduced SES [66-69].

The UK Index of Multiple Deprivation (IMD) score is a measure of SES, using a number of indicators (covering economic, social and housing metrics) to produce a deprivation score for each electoral ward in England [70]. In a cross-sectional study of patients with CKD carried out in Sheffield, patients were divided into quintiles of deprivation; living in the lowest SES quintile was associated with a lower eGFR at presentation, this was independent of socio-demographic, lifestyle and clinical risk factors [71].

There are numerous instruments for the measurement of QoL [72], all have strengths and limitations, as QoL is a highly subjective concept. The instruments may be symptom based, satisfaction based or organ system specific. The short form-36 (SF-36) consists of 36 questions covering well-being, functional status and perceptions of health status and it has been adapted for use in patients with renal disease (primarily aimed at those on maintenance dialysis) as the Kidney Disease Questionnaire (KDQOL) [73]. In a study of 205 patients with pre-dialysis CKD (stages 4 and 5) the KDQOL was administered; the mean scores obtained suggested that there was considerably impaired functional status compared to individuals with normal kidney function [74].

However, while the SF-36 and KDQOL are detailed assessment tools they are time-consuming to administer, to overcome this abbreviated tools have been devised. An example of this is the EQ5D [75] which was evaluated in a number of chronic disease groups and was found to perform well [76,77]. The EQ5D has also been used in health economic work to formulate quality adjusted life years [78]; it is for this reason that the EQ5D was chosen as the instrument for the assessment of QoL in RIISC participants.

### Cardiovascular assessment

#### Blood pressure measurement

Participants have their blood pressure (BP) measured using the BpTRU method after a five-minute rest. This is an oscillometric method that takes six consecutive readings and averages the last five measurements. This method has been shown to produce readings that are comparable to the daytime averages obtained by 24 hour ambulatory BP monitoring [79-83]. Routine clinic blood pressure readings may be inaccurate because of the absence of a prior rest, the single reading obtained or the environment in which the readings are taken. In a cohort of patients with CKD, BpTRU readings were significantly lower than routinely obtained clinic readings and correlated closely with 24-hour ambulatory BP daytime average readings (and 24-hour readings per se) [84].

### Arterial stiffness

A characteristic feature of arterial disease in CKD is thickening and calcification of the medial arterial layer, known as arteriosclerosis [17]. In its purest form, media calcification is concentric and does not extend into the arterial lumen. Increased collagen content, calcification, hyperplasia and hypertrophy of the vascular smooth muscle cells results in wall thickening and increased arterial stiffness. Although associations have also been established between the degree of arterial stiffness and atheromatous plaque burden [85], recent studies have failed to demonstrate a significant influence of traditional atherosclerotic risk factors on the development of arteriosclerosis [86], suggesting that alternative ‘non-atherogenic’ factors drive this process. There is certainly some overlap, however, as endothelial dysfunction and reduced NO bioavailability have been shown to contribute to arterial stiffening [87]. There is a strong association between arterial stiffening and mortality in CKD [17].

The pathophysiological effects of arteriosclerosis and arterial stiffening are best understood by an appreciation of the normal physiology of the aorta and large arteries. Their major functions are not only to deliver blood around the body but also to buffer the oscillatory changes in BP that result from intermittent ventricular ejection. The highly distensible arterial system ensures that most tissues receive near steady flow with no exposure to peak systolic pressures; this mechanism is so efficient that there is almost no drop in peripheral mean arterial pressure compared to the ascending aorta [88]. Loss of arterial distensibility results in a more rigid aorta that is less able to accommodate the volume of blood ejected by the left ventricle, resulting in greater pressure augmentation in systole and higher pulse pressures [89]. As arterial stiffness increases the loss of arterial distensibility exposes the myocardium, brain and kidneys to higher systolic pressures and greater pressure fluctuations arising from increased pulse pressures, resulting in myocardial and cerebral microvascular damage and an increased risk of heart failure, arrhythmia and stroke [90]. While the high systolic pressure increases LV afterload, lower diastolic pressure reduces diastolic coronary perfusion promoting ischaemia and placing greater reliance on systolic coronary perfusion [91,92].

Arterial stiffness is increased in patients with early stage CKD [17]. Aortic pulse wave velocity (PWV) is currently considered to be the “gold-standard” measurement of arterial stiffness [93]. Measures derived from central pulse-wave analysis (central systolic pressure, pulse pressure and augmentation index [AIx]) are considered indirect, surrogate markers of arterial stiffness and provide additional information on arterial wave reflections [93]. Increasingly, these markers are recognised as powerful predictors of cardiovascular mortality and morbidity in patients with CKD [17,93].

Theoretically, arterial stiffness should also lead to renal vascular damage and progressive renal impairment by similar mechanisms to those described above [17]. Three small studies have found an association; Taal *et al.* in 2007 used radial-dorsalis pedis PWV as a measure of arterial stiffness in 35 patients with advanced stage IV and V CKD and found PWV and AIx, predicted progression to ESKD [94]. In a Japanese study of 41 subjects with non-diabetic CKD AIx predicted a greater decline in renal function [95]. Interestingly, a subsequent study by this group in 42 patients failed to replicate this finding and did not demonstrate any relationship between PWV or AIx and progression of renal dysfunction [96]. A third study of 133 patients with stage III-IV CKD showed PWV to be a predictor of decline in renal function [97]. However, a larger study of 235 patients with CKD and longer follow-up failed to show any association between PWV and progression of CKD [51].

This latter study is in keeping with a prospective longitudinal analysis of the Framingham Offspring Study which did not find an association between baseline aortic PWV and incident CKD or microalbuminuria [98]. The differences between all of these studies highlight the lack of consistent data describing the natural history of the relationship between arterial stiffness and kidney disease and in particular the complex interactions between age, uraemia, blood pressure and medication in CKD patients [17]. Clearly larger longitudinal studies are needed to resolve this and interventional studies targeting arterial stiffness as a means of lowering cardiovascular events may then be warranted.

There are several commercially available systems for measuring PWV [99,100]. In this study we have chosen to use the Vicorder system. The Vicorder device has been developed to measure aortic PWV with little operator training and in a non-intrusive manner. It has been found to have very good intra- and inter-observer reproducibility in a number of conditions and produces comparable results to those obtained using the widely used method of applanation tonometry [101-103].

### Advanced glycation end products

The accumulation of advanced glycation end products (AGEs) is a putative promoter of endothelial dysfunction and the consequent increased cardiovascular risk experienced by individuals with CKD [104]. The accumulation of AGEs has been shown to correlate well with renal function and death in patients with CKD, dialysis patients and renal transplant recipients [37,104-106]. In a prospective cohort of 1700 patients with CKD stage 3, AGEs (as measured by skin autofluorescence) were independently associated with a number of traditional and non-traditional risk factors for CVD [37].

Advanced glycation end product levels will be measured by two complementary methods in RIISC. Firstly, AGE accumulation in the skin will be measured by skin autofluorescence (AGE reader™ [107]), secondly, serum concentrations of the AGE marker pentosidine will also be measured. In 2004 Meerwaldt *et al.* described a close correlation between skin autofluorescence and AGEs measured in skin biopsy samples in studies with both prevalent dialysis patients and those with persevered renal function (diabetic and non-diabetic subjects) [108], in 2005 they described a close correlation between AGEs and measures of inflammation (C-reactive protein) in a study of haemodialysis patients [105]. However, as several studies conducted in patients following kidney transplantation and in dialysis patients have not shown close correlations between AGEs measured in the skin (by skin biopsy rather than autofluorescence) and serum markers, we will explore the relationship between AGEs measured using both skin autofluorescence and the serum marker pentosidine [109-111].

All cardiovascular measurements are conducted in a room maintained at a constant temperature (22-24°C), using standardised operating procedures by trained personnel, at the same time of day (for each patient and at each time-point) and prior to phlebotomy and periodontal probing.

### Anthropomorphic assessment

Globally increasing cardiovascular mortality [112] together with the recognition of kidney disease as a cardiovascular risk factor [13] has led to greater interest in the relationship between obesity and kidney disease. A growing number of studies have concluded that adulthood obesity increases the risk for kidney disease [113].

Obesity is associated with a number of conditions known to increase the risk of CKD including hypertension, diabetes mellitus and heart failure [114]. Several studies have shown an association between adult obesity and CKD with approximately 25% of CKD in Western populations being attributable to obesity [106]. A recent study has also confirmed that a large proportion of the association between low socio-economic status and CKD can also be explained by obesity [115]. Studies looking at the relationship between fat distribution and CKD have produced conflicting results [113]. Furthermore, very few studies have examined longitudinally the relationship between obesity and progression of CKD [113]. Intriguingly, small studies in patients after bariatric surgery show improvements in blood pressure control, proteinuria and inflammatory markers as well as in GFR, although this last parameter needs to be interpreted with caution and confirmed in larger studies with harder end-points [116].

The current understanding of the biological mechanisms for the effects of obesity on CKD remains limited. Obesity may promote kidney damage directly through haemodynamic and hormonal effects or indirectly by favouring the development of diabetes and hypertension, and disorders with strong kidney involvement [113].

It has been postulated by Heitmann *et al.* that thigh circumference may be a cardiovascular risk factor in a prospective community based study of 2987 individuals. Decreased thigh circumference was related to increased risk of cardiovascular death and morbidity, this difference was independent of body mass index (BMI), percentage of body fat and waist circumference [117]. To date there is no published evidence that thigh circumference influences the progression of CKD or the CVD risk experienced by individuals with CKD.

RIISC participants have their height, weight, hip, waist and thigh circumference measurements taken using a standardised method (following the standard operating procedures included in the Additional file 1).

### Periodontal assessment

There is emerging interest in the potential association between chronic periodontal inflammation and endothelial dysfunction [44], this is based upon the hypothesis that atherosclerosis is an inflammatory disease and that chronic periodontitis contributes to the systemic inflammatory burden and thus potentiates atherosclerosis [118-120]. To further examine the relationship between periodontal health and Chronic kidney and cardiovascular disease, RIISC participants undergo a full mouth periodontal assessment which comprises: measurement of probing pocket depth (PPD), a measure of current disease status; recording of bleeding on probing (BOP), a measure of periodontal inflammation; clinical attachment loss (CAL), a measure of lifetime disease experience (carried out by a trained dental hygienist supported by a trained dental surgeon). Saliva samples are being collected for non-presumptive proteomic analysis and plaque samples are being collected for molecular microbiome analysis to address the hypothesis that the nature of the subgingival biofilm may correlate with renal status [121,122].

### Biomarkers

There are a number of biomarkers that have been associated with CKD. These include: (i) markers of renal impairment; (ii) risk factors for CVD; and (iii) risk factors for progressive CKD. To date, some studies of renal biomarkers have been limited by methodological shortfalls (the methods used for measuring renal progression, the large number of biomarkers studied and the exclusion of certain groups of patients). Table 5 describes the index biomarkers selected in the RIISC study and the current evidence of their possible role in the progression of CKD.

The biomarkers listed in Table 6 have all been identified as being associated with progressive CKD in human studies of at least 50 patients, there are a number of other putative biomarkers (such as pro-inflammatory cytokines and vitamin D isotypes) where such evidence does not currently exist but where early experimental work suggests a plausible link with renal progression, RIISC aims to clarify the role that these biomarkers have (alone or in combination with each other) in the progression of CKD.

**Table 6:** Biomarkers measured as part of the RIISC protocol

**Biomarker**	**Patho-physiological basis**	**Number of patients**	**Definition progression**	**Evidence to date**
Cystatin C	Marker of kidney function [123]	117	Doubling serum creatinine or ESKD	Cystatin C predicted renal decline (doubling of Creatinine or arrival at ESKD) in the MMKD study [124]
Neutrophil Gelatinase-Associated Lipocalin (NGAL)	Marker of tubulo-interstitial injury [125]	96	Doubling serum creatinine	Serum and urine NGAL was associated with renal decline (doubling of serum creatinine) [126]
Asymmetric Dimethylarginine (ADMA)	Marker of endothelial dysfunction [127]	225	Increased proteinuria, rate of change of eGFR	A study of 225 diabetics found that ADMA was associated with renal progression (increase in proteinuria, rate of change of eGFR) [128]
		227	Doubling serum creatinine or arrival at ESKD	
		131	Arrival at ESKD	ADMA levels above the median were more likely to reach an endpoint [129]
				ADMA was an independent risk factor for renal progression [130]
B-type Natriuretic protein (BNP)	Marker of cardiovascular dysfunction [131]	227	Doubling serum creatinine or arrival at ESKD	Elevated BNP and pro BNP were associated with progression to end points [132]
		382	Arrival at ESKD	BNP correlated strongly with risk of mortality but not progression of CKD [38]
Homocysteine (Hcy)	Marker of endothelial dysfunction [133]	316	Development of albuminuria from normoalbuminuria	Hyperhomocysteinaemia was a predicted the development is albuminuria [134]
C-reactive protein (CRP)	Marker of inflammation	804	Rate of change of eGFR	Neither serum CRP or leptin predicted renal progression [135]
Adiponectin	Marker of metabolic disturbance [136]	1330	Arrival at ESKD	The group of patients with microalbuminuria who progressed to ESRF had higher adiponectin levels [137]
Free light chains (FLCs)	Marker of renal function and possible inflammation [138]	282 healthy controls, 772 South Asian diabetics, 91 Caucasian diabetics	Development of microalbuminuria	Elevated serum FLCs were a risk factor for the development of microalbuminuria [138]
Fibroblast growth factor 23 (FGF 23)	Marker of metabolic disturbance [139]	227 non-diabetics with normal renal function and CKD (GFR>60 = 121, GFR<60 =106	Doubling serum creatinine or arrival at ESKD	Both c-terminal and intact FGF23 independently predicted progression of CKD after adjustment for age/gender/GFR and proteinuria [139]
Urinary MCP1	MCP-1/CCL2 is a chemokine which is upregulated in CKD [140,141]	215 patients with CKD undergoing a renal biospy	Doubling of serum creatinine or arrival at ESKD	ACR, urinary MCP-1 and interstial macrophage numbes were interdependent. ACR, macrophage numbers chronic damage and creatinine predicted renal survival [142]

MMKD- mild to moderate kidney disease study.

The appropriate collection and handling method of samples for biomarker analysis is important as many putative biomarkers are unstable and degrade rapidly from biological samples; it is accepted that this may limit their wider clinical application and to address this concern a sub-study investigating the reproducibility and stability of certain biomarkers will be carried out. As part of the RIISC protocol all samples are handled as described in Additional file 1: Appendix 7.

### Genetic analysis

The influence of genetic factors on CKD progression has yet to be elucidated; in one study no relationship was found between several single nucleotide polymorphisms (SNPs) and progressive CKD [136]. When a genome wide association study (GWAS) was performed a gene related to uromodulin was shown to be associated with renal function, although its relationship to renal progression has yet to be studied [143]. In a study of dialysis patients, patients with “mild” CKD and a group of healthy controls, polymorphisms of genes that influence endothelial function were explored; the authors reported that some genotypes were found more frequently in some diagnostic groups than others; this is an interesting area for future work [144].

In an analysis of nine cohort studies, containing over 23 000 participants, a GWAS was performed; serum creatinine, eGFR and cystatin C were used as measures of renal function [145]. There were 109 SNPs associated with serum creatinine; these were distributed over five loci, only one of these had previously been described as having an association with kidney function. When potential associations between the loci and eGFR or cystatin C were investigated two of the four loci were associated with eGFR but not cystatin or CKD, none of the four novel loci were associated with weight, hypertension or diabetes [145].

In another large GWAS study over 130 000 individuals were included; the aim of the study was to stratify participants by four key risk factors, hypertension, age, gender and diabetes to identify novel loci [146]. Six new loci were identified that were associated with low eGFR, there was variability with some loci being more pronounced in younger patients and some being more frequent in certain ethnic groups [146].

Multiple SNP analysis and GWAS will be performed to further explore these potential associations.

Data collection and analysis.

The aim of RIISC is to recruit a minimum of 1000 participants. This will allow robust interpretation of the relationship between the variables that will be under study in the cohort and their relationship to clinical outcomes. Data collected is stored in a specially designed database that allows detailed recording of the demographic and phenotypic characteristics of the cohort across multiple sites; data can be rapidly retrieved and analysed.

The data collected will used to assess the generalizability of existing renal risk scores, such as the one devised by Tangri *et al.* in 2011, and can be used to generate a new renal risk score [147] The purpose of such scoring systems is to aid the clinician in risk stratification of patients with CKD, this allows the patients at highest risk of progression and adverse cardiovascular outcomes to receive targeted treatment while those at lower risk can be reassured and provided with lifestyle advice [148]. Current risk scores have focused on traditional risk factors as there has been insufficient data on which of the non-traditional risk factors are implicated in progression; the RIISC cohort aims to provide such evidence and this will be utilised in risk score development. From the work on non-traditional risk factors conducted thus far it seems probable that combinations of biomarkers will be required for risk score development rather than individual bio-markers [125,149].

Predictive algorithms will be developed which could be applied in clinical practice to estimate risk prospectively. We will be guided by the general approach described by Harrell and colleagues [150], potential predictors of outcome will be identified from the existing literature and from the cohort. We will develop appropriate prognostic models based upon Cox constant proportional hazard models. We will examine the linearity of response to each continuous variable in the model, and examine whether transformations (pre-specified) or more complex restricted cubic splines, improve the model fit using a sequential model building strategy based upon Akaike’s Information Criterion Final models will be selected using backwards stepwise selection [150].

## Discussion

Chronic kidney disease is a significant cause of morbidity and mortality but the natural history of progression is not clear. Some patients are at substantial risk of progression to end stage renal disease and while some risk factors are well known (proteinuria, diabetes and hypertension) it is probable that there are other prominent novel risk factors that influence progression [151-154].

There have been a number of biomarker studies aiming to identify non-traditional risk factors; however these have been limited by methodological shortfalls such as small study size, short follow up periods and the failure to measure the proposed biomarker at multiple time-points. Another limitation is that some studies aimed to simply identify bio-markers that were associated with CKD (so these may be markers of kidney function rather than markers of renal disease progression or cardiovascular risk), those which did consider renal disease progression were limited by the endpoints employed, arbitrary changes in serum creatinine between two time points and/or progression to ESKD.

The clinical management of patients with CKD is based on a number of clinical guidelines (e.g. NICE, SIGN, and KDIGO), these cover the identification of CKD, referral criteria to nephrology services and guidance on the management of complications like renal bone disease and anaemia, with little focus on assessing and managing the risk of CKD progression [47,155,156]. The RIISC cohort is comprised of patients who are at highest risk of progressive CKD and adverse cardiovascular outcomes that consequently fulfill the criteria for secondary care follow-up according to these guidelines. The principle of this rolling recruitment cohort study is important, as it will be the first cohort of high risk CKD secondary care patients who undergo detailed bio-clinical assessment at sequential time-points with prolonged follow up. As such, this will allow us to assess how the contemporary management of CKD might influence the natural history of the disease.

The repeated assessment of RIISC participants also sets it apart from other cohort studies; only the R^2^ID and CRISIS cohorts include repeated measures of vascular health and blood and urine collection, though the patients studied are from different CKD populations [157]. While it seems likely that the natural history of CKD may change with time (especially in the light of targeted, guideline-led management), in a recent review (aimed at determining whether early referral of patients with CKD was cost effective) Black *et al.* commented in 2010 that while there is evidence that individuals referred to nephrology services had slower rates of decline, this was likely related to more aggressive control of blood pressure, although there were “significant evidence gaps about how to best manage people with CKD” [158].

The RIIISC methodology has been designed with the aim of addressing some of these evidence gaps but we appreciate that there are a number of aspects of the study design, and omissions from it, which could reduce the clinical relevance of the data produced, these aspects (and the rationale for them) are summarised in Table 7.

**Table 7:** RIISC protocol; areas of controversy

**Omission**	**Rationale**
No gold standard measure of kidney function used for either screening or renal progression	While inulin and iohexol clearance are the gold standard measures of kidney function, radioisotope methods are accepted as they are easier and less expensive [159]. However these are still invasive and costly and would increase the burden on potential and actual participants. The MDRD equation with IDMS traceable creatinine results was chosen because it is part of routine clinical practice (thus making our cohort representative of the CKD population). The application of other creatinine-based equations (e.g. CKD EPI) will also be explored.
No dietary restrictions placed upon patients prior to clinic attendance	Serum creatinine is affected by diet and meat consumption prior to testing can influence the result obtained [53]. In some studies participants are asked to refrain from eating meat in the 24 hours preceding testing [37], however we decided that this placed an additional burden on patients and would make results obtained is not generalisable to routine clinical practice.
No cardiac imaging (CT or echocardiography)	While coronary calcification has been described in CKD and detailed cardiac imaging has been conducted as baseline in some cohort studies; this is invasive and adds complexity to the protocol. The non-invasive measures of arterial stiffness have been shown to correlate well with more invasive methods [32,93,160].
No use of Dexa scanning to measure bone health	Patients with CKD are known to be at risk of bone loss and fractures, renal bone disease is also a risk factor progression and cardiovascular events [161]. Dexa scanning is the gold standard measurement of bone density but novel biomarkers of bone turnover, such as FGF 23, have been shown to be associated with progressive CKD and cardiovascular risk, without radiation exposure and at lower cost and inconvenience to the participant [162].
The use of a short quality of life questionnaire that is non-renal specific	There are a number of renal specific quality of life measures available, they vary in detail but tend to focus on symptom burden specific to the renal population. The SF 36 is a generic questionnaire that has been validated in CKD, though there is no evidence that using it in combination with the KDQOL questionnaire is additive [77,163,164]. There is evidence that the EQ5D in combination with the KDQOL provide complementary information on patient perception of disease; however even the abbreviated the KDQOL contains 36 questions (some being very detailed) and would be difficult to complete for patients who do not speak English as a 1^st^ language (as many of the RIISC cohort may not) [77,163].
The recruitment of patients from secondary care only	The majority of CKD is managed in the community (primary care) [165] and that the data obtained from this, higher risk, cohort may not be applicable to primary care patients. However the focus on RIISC is specifically on those patients at highest risk of progression to ESKD and under secondary care follow-up; that is those patients who have the highest disease burden.

If progressive CKD and the attendant cardiovascular risks are propagated by systemic inflammation and endothelial dysfunction then strategies to reduce inflammation and endothelial dysfunction could be beneficial. Beyond management of traditional risk factors (control of hypertension and diabetes, smoking cessation and lipid lowering) there is little evidence for other interventions to date. However the overlap between traditional and novel risk factors via common inflammatory pathways may direct future therapies with a focus on pro-inflammatory targets [166]. In order to target intervention at novel risk factors then a clear understanding of those risk factors is required. The aims of the RIISC study are to clarify the influence of novel risk factors in the progression of CKD and as a consequence to identify patients at highest risk of adverse outcomes in order to target any intervention aimed at reducing that risk.

## Conclusions

The RIISC cohort comprises a cohort of well-defined patients with high risk CKD; the cohort will undergo detailed cardiovascular, renal and inflammatory phenotyping. The protocol has been designed to ensure that accurate and reproducible data are produced and recorded for each participant at each time-point. The potential changes in the natural history of progressive CKD over time will be examined by repeated phenotyping during the study period and prolonged follow up with robust end-points and outcomes. The RIISC study should contribute to increasing our understanding of the mechanisms associated with the increased risk seen in people with progressive CKD, and identify targets for new therapies.

## Abbreviations

CKD: Chronic kidney disease; eGFR: Estimated glomerular filtration rate; RIISC: Renal impairment in secondary care; CVD: Cardiovascular disease; ESKD: End stage kidney disease; RCT: Randomised control trials; CRIB: Chronic renal impairment in Birmingham; MMKD: Mild to moderate kidney disease; LCKD: Longitudinal chronic kidney disease; CRISIS: Chronic renal impairment in Salford; CRIC: Chronic renal insufficiency cohort; SEEK: Study for the evaluation of early kidney disease; R2ID: Renal risk in Derby; NICE: National institute of clinical excellent; ACR: Albumin creatinine ratio; BP: Blood pressure; PWV: Pulse wave velocity; PWA: Pulse wave analysis; AGEs: Advanced glycation end products; BoP: Bleeding on probing; PPD: Probing pocket depth; CAL: Clinical attachment loss; QoL: Quality of life; MDRD: Modification of diet in renal disease; SES: Socio-economic status; IMD: Index of multiple deprivation; SF-36: Short form 36; KDQOL: Kidney disease quality of life; AIx: Augmentation index; BMI: Body mass index; NGAL: Neutrophil gelatinase-associated lipocalin; ADMA: Asymmetric dimethylarginine; BNP: B-Natiuretic peptide; Hcy: Homocysteine; CRP: C-reactive protein; FLCs: Free light chains; FGF 23: Fibroblast growth factor 23; SNP: Single nucleotide polymorphisms; GWAS: Genome wide analysis study.

## Competing interests

None of the authors have any competing interests.

## Authors’ contributions

SS; designed the study, completed the applications process, was involved in patient recruitment and wrote the manuscript. PS; designed the periodontal aspect of the study, carried out the periodontal assessments and reviewed the manuscript. MD; was involved in the application for ethics and R&D approval, prepared the standard operating procedures and is involved in patient recruitment. MJ; was involved in the recruitment of patients and the on-going ethics and R&D process. KN; was involved in the recruitment of patients and the on-going ethics and R&D process. OK; was involved in the preparation of sample handling protocol and was involved in the collection of samples for biomarker analysis. IC; designed the periodontal aspect of the study and reviewed the manuscript. TD; designed the periodontal aspect of the study and reviewed the manuscript. CF; was involved in the study design, recruitment of patients and contributed to writing the manuscript. PC; is the primary investigator, was involved in the design of the study, recruitment of patients and was involved in writing and reviewing the manuscript. All authors read and approved the final manuscript.

## Pre-publication history

The pre-publication history for this paper can be accessed here:

http://www.biomedcentral.com/1471-2369/14/95/prepub

## Supplementary Material

Additional file 1 **Appendices.** Standard operating procedures (SOPs Appendix 1. Blood pressure measurement using the BpTRU device [1]. Appendix 2. Measurement of arterial stiffness using the Vicorder device [2]. Appendix 3. Measurement of advanced glycation end products using the AGE reader device [3]. Appendix 4. Measurement of weight [4]. Appendix 5. Measurement of height [4] Appendix 6. Measurement of waist/hip and thigh circumference [4,5]. Appendix 7. Plasma, serum and urine sample handling/processing [6,7]. Appendix 8. Collection of samples for genetic analysis [8]. Appendix 9. Urinalysis [9]. Appendix 10. Periodontal assessment [10]. Appendix 11. Plaque collection [11]. Appendix 12. Saliva sample collection. Appendix 13. Demographic data questionnaire. Appendix 14. The EQ5D tool for assessment of quality of life, used with permission from the EuroQoL group [12]. Click here for file
