# Supplementary material for: The natural history of, and risk factors for, progressive Chronic Kidney Disease (CKD): the Renal Impairment in Secondary care (RIISC) study; rationale and protocol
Source: BMC Nephrol. 2013 Apr 25;14:95. doi: 10.1186/1471-2369-14-95 (PMC3664075; doi:10.1186/1471-2369-14-95)
Supplement: Additional file 1 — Appendices. Standard operating procedures (SOPs Appendix 1. Blood pressure measurement using the BpTRU device [1]. Appendix 2. Measurement of arterial stiffness using the Vicorder device [2]. Appendix 3. Measurement of advanced glycation end products using the AGE reader device [3]. Appendix 4. Measurement of weight [4]. Appendix 5. Measurement of height [4] Appendix 6. Measurement of waist/hip and thigh circumference [4,5]. Appendix 7. Plasma, serum and urine sample handling/processing [6,7]. Appendix 8. Collection of samples for genetic analysis [8]. Appendix 9. Urinalysis [9]. Appendix 10. Periodontal assessment [10]. Appendix 11. Plaque collection [11]. Appendix 12. Saliva sample collection. Appendix 13. Demographic data questionnaire. Appendix 14. The EQ5D tool for assessment of quality of life, used with permission from the EuroQoL group [12]. [file 1471-2369-14-95-S1.docx]

# Appendices: Standard operating procedures (SOPs)

## Appendix 1: Blood pressure measurement using the BpTRU device([1](#_ENREF_1))

Purpose

To obtain blood pressure readings on patients in the RIISC study which are consistent with the study protocol.

All participants will have their blood pressure recorded at all time-points

Preparation and Method

Patients will have rested in a quiet room for 5 minutes prior to taking a measurement.

Patients will have the monitor sited at the same level as their heart with their back and arm supported in a relaxed position. Both feet should be flat on the floor.

They will be asked not to talk while the recording is taking place.

Align the artery indicator on the cuff with the patient’s brachial artery. Wrap the cuff around the arm and check that the white index marking on the edge of the cuff falls within the white range markings on the inside surface of the cuff.

If the index does not fall within the range markers, replace the cuff with a smaller or larger size.

Ensure the cuff is tight but allow two fingers to be inserted between cuff and arm.

Taking a BP measurement.

Turn on machine or press the Clear button to clear memory between patients.

Attach cuff to upper arm of patient

Use the cycle button to select an automatic series of measurements (indicated by a character from 1-5 in the Cycle display.)

Press the BP start button to begin the measurement. (Wait 5 seconds after turning on the BpTRU before pressing the start button.)

Press the Stop button at any time to stop the measurement and deflate the cuff or to pause between measurements.

Results

A tone will sound at the completion of six measurements.

After 5 seconds the reading display will show “A” and the average readings of the last 5 measurements is displayed.

## Appendix 2: Measurement of arterial stiffness using the Vicorder device([2](#_ENREF_2))

Purpose

This SOP describes procedures to ensure the correct use of the Vicorder Equipment for the RIISC study to obtain measurements which are consistent with the study protocol.

All participants will have their pulse wave velocity and pulse wave analysis measured at all time-points

Method

Vicorder readings will be recorded at all study time points: baseline, 6 months, 18 months, 3 years, 5 years and 10 years.

Take 3 readings; if there is a more than 10% deviance from expected normal of 7m/s; continue to take readings until there are two within 10% of one another. If the first three readings are above 12m/s then take another three readings.

Note which leg and arm used for readings and enter data. Use same arm and leg throughout study at all time points. If at any time point this is different, record reason for change.

Ensure room temperature kept between 22 and 24 degree Celsius: use temperature log sheet to record.

Ensure that all data collected is stored in spreadsheet.

## Appendix 3: Measurement of advanced glycation end products using the AGE reader device([3](#_ENREF_3))

Purpose

The purpose of this SOP is to ensure the correct use of the AGE reader Equipment for the RIISC study

The AGE Reader CU™ is a proprietary device that can non-invasively assess the tissue accumulation

of Advanced Glycation End products (AGEs) and obtain measurements that are consistent with the study protocol.

All participants will have their AGEs measured at all time-points

Intended Use

Measurements should be done on the dominant arm on healthy undamaged skin

without birthmarks or excessive hair growth, tattoos or scars. Self tanning agents must not be used for at least 2 days. If patient has used self tanning agents document and inform the patient not to use next time 2 days before the appointment. Sun-blockers and other skin care products should be removed before measurement.

Pigmented skin

The device and its software have been validated in patients with Fitzpatrick class 1-4 skin colour. For measurements on patients with Fitzpatrick class 5-6 (dark brown or black), users should check with the manufacturer or distributor for the correct software version in order to avoid unreliable results. If a measurement is performed on a skin type that is too dark to give a reliable result, the AGE Reader CU will give a warning.

UV-Radiation

Using the guidelines of the ICNIRP it is concluded that during AGE Reader CU measurements, as intended, even when repeated up to a 100 times on the same skin site within an 8-hour period, the local radiation exposure on the skin of the patients, and to the eyes of patients and operators remain considerably below the maximum allowed values for that period. Radiation exposure to the eyes normally does not occur. Exposure of the eyes longer than 60 seconds per 8-hour period should be avoided (ie do not look directly into the UV light)

Procedure and method

Follow the instructions as set out in AGE reader operator manual 2010 to be found with equipment([3](#_ENREF_3)).

## Appendix 4: Measurement of weight([4](#_ENREF_4))

Purpose

The purpose of this SOP is to ensure that all weights recorded are accurate, reproducible and consistent with protocol requirements.

All participants will be weighed at all time points

Preparation and Method

Place a clean paper towel on footplate of scales.

Measure weight with the participant wearing skirt or trousers and shirt, but no jacket or jersey and no shoes.

Place the scales on a hard floor. If there is no hard surface available, place the scales onto the wooden board, on the floor. Reset the zero button, be sure the scales measure in kilograms. When the zero shows ask the participant to step on, without hesitation, and then read off the flashing answer, and record value.

Sitting weight will be recorded on patients who cannot stand but on mechanical scales and only if safe to transfer patient with assistance.

## Appendix 5: Measurement of height([4](#_ENREF_4))

Purpose

The purpose of this SOP is to ensure that all height readings obtained for the purposes of the RIISC study are accurate, reproducible and consistent with protocol requirements.

The height of all participants is recorded at all time-points

Preparation and method

Participant to remove shoes and to stand with feet together, flat on the base plate and with heels against the back of the plate, and to stand as tall as possible. Arms should be held loosely at the side. Tilt the head to the Frankfort plane position, so that an imaginary line passing through the external ear canal and across the top of the lower bone of the eye socket immediately under the eye would be parallel to the floor (i.e. horizontal). Check the position by holding the Frankfort plane card beside the participant’s face. Ask the participant to take a deep breath in, re-check the Frankfort plane position and bring the headpiece down on the centre of the participant's head and check the level using the spirit level. Take the reading to the nearest 1 cm and record.

## Appendix 6: Measurement of waist/hip and thigh circumference([4](#_ENREF_4), [5](#_ENREF_5))

Purpose

To obtain measurement of waist, hip and thigh circumference measurements for the purpose of the RISC study that are accurate, reproducible and consistent with protocol requirements.

All participants will have their waist, hip & thigh circumference recorded at all time-points

Preparation and method

Ask the participant to face you and to stand straight with feet together and looking straight ahead. Stand to the right of the participant. Hold the tape in your right hand with the side of the tape where the scale begins facing you. Pass the other end of the tape round the back flank with your left hand and ask the participant to hold it whilst you retrieve the end of the tape from his/her left hand.

This should leave you standing slightly to the participant's left when you draw the tape taut.

Waist circumference

Make two marks with a waterproof pen at the costal margin (lower rib) and the iliac crest. Apply tape at a point midway between these two points, in line with the mid axilla. Measure on the skin if possible.

Ensure that the tape is horizontal.

Ask the participant to breathe out gently and to look straight ahead (to prevent them from contracting their muscles or holding their breath). Pull tape taut and measure to the nearest cm at the end of a normal expiration and record value. If participant is tense, repeat the measurement and take the new reading if it is higher.

Hip circumference

Locate the greater trochanter (this will be at the widest part of the hips, at the level of the buttock line). To check the levels you have to position the tape on the right flank and peer round the participant's back from their left flank to check that it is level.

While measuring ask participant to breathe out gently, to let arms hang loosely by their sides and to look straight ahead (to prevent them from contracting their muscles or holding their breath). Pull tape taut and measure to the nearest cm and record value on the questionnaire. Try to take the measurement (to the nearest cm) in mid-expiration when the abdominal muscles are maximally relaxed. If participant is tense, repeat the measurement and take the new reading if it is higher.

Waist and hip circumference should all be measured on the skin if participant consents and it should be recorded if this does not happen.

Thigh Circumference

Pass a measure immediately below the gluteal fold of the right thigh. Measure to the nearest cm. Ensure that the same leg is used for all measurements in the study. Note left or right on the database.

## Appendix 7: Plasma, serum and urine sample handling/processing([6](#_ENREF_6), [7](#_ENREF_7))

Purpose

The purpose of this SOP is to ensure standardised operating procedures, when collecting blood and urine samples for the purpose of this study.

Blood, urine and saliva samples will be collected from all participants at all time-points

Introduction/Method

1. Collect blood samples using vacutainers (order of draw: 2 x red, 1 x EDTA, 1 x Paxgene)
2. Tubes should be completely filled by the vacuum in order to obtain the correct ratio of blood to additive. Over and under filing alters the ration and changes results.
3. Thoroughly mix by inverting the tube 8-10 times
4. Leave serum (2 x red top) to clot for 1 hour at room temperature
5. Spin at 2500rpm for 10 minutes at 4°C
6. Spin the EDTA samples immediately at 2500rpm for 10 minutes at 4°C
7. Urine collected as midstream clean catch. Where possible ask the patient to provide a fresh sample. Urine samples collected more than 2 hours ago should be discarded.
8. Spin at 3000rpm for 15 minutes at 4°C
9. After spinning of all samples aliquot and transfer to a -80°C freezer

## Appendix 8: Collection of samples for genetic analysis([8](#_ENREF_8))

Purpose

The purpose of this SOP is to ensure standardised operating procedures when collecting and processing DNA samples for the purpose of this study. Applies to samples collected using PAXgene Blood DNA Tubes ONLY.

Samples for DNA extraction are collected at baseline only

Introduction

Method

Blood collection:

Ensure that the PAXgene Blood DNA Tube is at room temperature (18-25°C) prior to use.

The PAXgene Blood DNA Tube should be the last tube drawn.

Under-filling of the tubes will result in an incorrect blood-to-additive ratio and may lead to incorrect analytical results or poor product performance.

Since the PAXgene Blood DNA Tube contains a chemical additive, precautions should be taken to prevent possible backflow from the tube during blood drawing.

Transfer by a syringe is not recommended.

Following draw, gently invert the PAXgene Blood DNA Tube 8-10 times.

Store the PAXgene Blood DNA Tube upright at room temperature until freezing at -20°C.

Guidelines for Freezing PAXgene Blood DNA Tubes containing blood samples:

To freeze PAXgene Blood DNA Tubes, stand them upright in a wire rack. If wire rack is not available, freeze horizontally in a plastic bag. Do not freeze tubes upright in a Styrofoam tray as this may cause the tubes to crack.

Blood samples collected using PAXgene Blood DNA Tubes can be stored at 15-25°C for up to 14 days, at 2-8°C for up to 28 days, or at -20°C for up to 3 months. For long-term storage, freezing the samples at -70°C is recommended.

If tubes are to be stored for no longer than 10 weeks, freeze the tubes in the wire rack at -20°C. For longer storage periods, freeze the tubes first at -20°C for 24hrs, and then transfer them to -70°C or -80°C.

Thaw PAXgene Blood DNA Tubes in a wire rack at ambient temperature (18-25°C) for approximately 2 hours or at 37°C in a water bath for approximately 15 minutes. After thawing, carefully invert the tube 10 times. Store the tubes on ice until you are ready to begin the PAXgene DNA purification procedure.

## *Appendix 9:* Urinalysis([9](#_ENREF_9))

Purpose

The purpose of this SOP is to ensure that urinalysis conducted as part of the RIISC study is accurate, reproducible and consistent with protocol requirements.

All participants will have urinalysis will be recorded at all time-points

Method

Participants will be asked to provide a urine sample on arrival at the clinic.

Samples will be fresh or no more than 2 hours old.

Samples must be at room temperature.

Women will be asked if they are menstruating.

Turn on analyzer.

Use gloves and apron.

Select Strip Test from Select Ready screen.

Remove a reagent strip from the bottle and quickly replace the cap.

Select Enter New Patient from Patient Information screen.

Enter patient study number.

Select Start from prepare test screen.

Complete following step in 8 seconds:

Dip strip in urine sample. Wet all pads.

Quickly remove strip from urine.

Drag the edge of the strip against the side of the sample container as you remove it.

Blot by touching the edge of the strip to a paper towel to remove excess urine.

Place strip in channel of table with pads facing up.

Slide or push strip to end of the channel.

After 8 seconds the instrument pulls in the test table.

When test completes, the Results screen displays.

Select More to view the second screen of results.

Select print.

Select done to return to the Select Ready screen.

Discard the reagent strip.

Wipe the table insert with damp tissue between tests to remove any urine residue.

## Appendix 10: Periodontal assessment([10](#_ENREF_10))

Purpose

The purpose of this SOP is to ensure reproducible and accurate diagnosis of the severity and extent (both current and historic) of periodontal disease.

Participants will undergo a periodontal assessment at baseline, 36, 60 and 120 months.

Method

1. Explain the procedure to the patient.
2. Enquire about any medical conditions (such as a history of infective endocarditis) and medications (such as warfarin) that may render a detailed periodontal examination unsafe.
3. A trained dental professional conducts a general oral examination and notes any missing teeth.
4. Periodontal measurements are carried out on all teeth present using *the UB-WHO-CF15* constant-force periodontal probe (Implantium.co.uk).
5. For each tooth, record both probing depth and recession on the mesial and distal aspects of the buccal and palatal/lingual surfaces. So for each tooth, record 4 sets of periodontal measurements (proximal sites).
6. The Probing Depth is measured to the nearest millimetre from the base of the periodontal pocket to the gingival margin.
7. The Recession is measured to the nearest millimetre from the cement-enamel junction (CEJ) to the gingival level. If the gingival level is at the CEJ, the recession is recorded as 0mm, if the gingival level is apical to the CEJ, the recession is recorded as a positive integer and if the gingival level is coronal to the CEJ, the recession is recorded as a negative integer.
8. The total Clinical Attachment Loss (CAL) is recorded as the sum of the probing depths and recession (either 0 or positive or negative)
9. Clinical Attachment Loss = Probing Depth + Recession
10. On completion of these measurements for a dental quadrant, a dichotomous record of Bleeding on Probing (BoP) is recorded (either present or absent) for each site probed. This represents bleeding from the base of the pocket.

## *Appendix 11*: Plaque collection ([11](#_ENREF_11))

Purpose

The purpose of this SOP is to ensure standardised operating procedures, at all sites, when collecting and processing subgingival plaque samples for the purpose of this study.

All participants will have plaque collected at baseline, 36, 60 and 120 months.

Introduction

The objective is to sample subgingival plaque from up to 6 ‘representative sites’ for a patient with periodontitis and 3 sites for a patient with health or gingivitis. These sites should be as distant as possible from each other, one per sextant, if possible.

Method

1. Identify ‘deep’ (≥ 6mm) pockets, if any, using detailed periodontal charting.
2. For patients with periodontitis, select 3 deep and 3 shallow (≤3 mm) pockets for sampling. For such patients with less than 3 deep pockets, select as many deep pockets and the rest as shallow pockets. Similarly for patients with less than 3 shallow pockets, select as many deep pockets and the rest as shallow pockets.
3. For healthy patients or patients with gingivitis, select 3 shallow pockets (mesio-buccal).
4. For such patients with less than 6 eligible pockets, select as many as possible.
5. Once representative teeth have been identified, they should be isolated using cotton wools rolls.
6. Supragingival plaque should be removed using a cotton wool pledget and the teeth air dried
7. Four size 40 paper points are introduced simultaneously to the selected pockets and left in situ for 10 seconds
8. The paper points are then removed and placed in a cryotube with a yellow lid containing Tris buffer and stored separately as either ‘deep pocket samples’ or ‘shallow pocket samples’ depending on the classification of the site sampled.
9. Samples to be stored in a -80°C freezer. (Store grouping samples of the same subject no.)
10. Batch send to Birmingham Dental Hospital, marked FAO Prof Iain Chapple/Prof Thomas Dietrich, on study completion.

## *Appendix 12*: Saliva sample collection

Purpose

The purpose of this SOP is to ensure standardised operating procedures, at all sites, when collecting and processing saliva samples for the purpose of this study.

All participants will have saliva collected at all time-points.

Method

1. Ask patient if they have followed pre-sampling instructions with respect to eating, drinking, smoking and brushing teeth prior to sampling appointment.
2. Instruct the patient to rinse mouth with sterile water to remove food residue before sample collection. Wait at least 10 minutes after rinsing before collecting saliva to avoid sample dilution.
3. Give the patient the sterile saliva sampling marble.
4. Label the saliva sample tube (graduated Falcon tube) with study no. study time-point, date and time.
5. Remove lid from the saliva sample tube and place saliva sampling funnel into the saliva sample tube.
6. Place the combination of the saliva sampling funnel and the saliva sample tube into a cup of ice. Give to the patient to hold.
7. Instruct the patient to place a sterile marble in their mouth and continually roll it around for 5 minutes.
8. Instruct the patient to retain the marble in their mouth while expectorating the resulting saliva.
9. Time the patient for 5 minutes and ensure that a minimum of 1.0ml of saliva has been collected.
10. If 1.0ml of saliva has not been collected in 5 minutes then have the patient continue until 1.0ml has been collected.
11. Record the time it took to reach 1.0ml
12. If the patient accidentally spits the marble into the funnel, they can retrieve it with their fingers and replace in it their mouths.
13. Take the apparatus from the patient. Remove the funnel. Leaving the sample tube in the cup of ice, place the lid on.
14. Take the saliva sample to the laboratory.
15. Record samples receipt
16. Record total volume. Calculate flow rate (ml/min).
17. Samples are centrifuged at 2500rpm for 10 min at 4 °C to remove debris.
18. Transfer the supernatant into a microcentrifuge tube. Take care not to disturb the pellet. (Store a maximum of 1.8ml.)
19. Label with study no. and study time-point (with labels provided and by hand).
20. Snap freeze the supernatant in liquid nitrogen.
21. Transfer to a -80°C freezer for storage.

## *Appendix 13:* Demographic data questionnaire

| **DOB** |  |
| --- | --- |
| **Study Number** |  |
| **Country of Birth** |  |
| **Ethnicity** |  |
| **Post Code** |  |
| **Year at Address** |  |
| **Highest qualification** | None |
|  | GCSE/ O’ level |
|  | NVQ |
|  | A’ level |
|  | Undergraduate |
|  | Post graduate |
| **Currently Employed** | Yes |
|  | No |
|  | Retired |
| **Job** | None |
|  | Unskilled/manual |
|  | Skilled/manual |
|  | Clerical |
|  | Managerial |
|  | Professional |

## Appendix 14: The EQ5D tool for assessment of quality of life, used with permission from the EuroQoL group([12](#_ENREF_12))

By placing a tick in one box in each group below, please indicate which statements best describe your own health state today.

**Mobility**

I have no problems in walking about ❑

I have some problems in walking about ❑

I am confined to bed ❑

**Self-Care**

I have no problems with self-care ❑

I have some problems washing or dressing myself ❑

I am unable to wash or dress myself ❑

**Usual Activities** (*e.g. work, study, housework, family or*

*leisure activities)*

I have no problems with performing my usual activities ❑

I have some problems with performing my usual activities ❑

I am unable to perform my usual activities ❑

**Pain/Discomfort**

I have no pain or discomfort ❑

I have moderate pain or discomfort ❑

I have extreme pain or discomfort ❑

**Anxiety/Depression**

I am not anxious or depressed ❑

I am moderately anxious or depressed ❑

I am extremely anxious or depressed ❑

9 0

8 0

7 0

6 0

5 0

4 0

3 0

2 0

1 0

100

Worst

imaginable

health state

0

Best

imaginable

health state

To help people say how good or bad a health state is, we have drawn a scale (rather like a thermometer) on which the best state you can imagine is marked 100 and the worst state you can imagine is marked 0.

We would like you to indicate on this scale how good or bad your own health is today, in your opinion. Please do this by drawing a line from the box below to whichever point on the scale indicates how good or bad your health state

**Your own**

**Health state**

**Today**

**References**

1. Division BMD. BpTRU operator's manual. 2007 [updated 200716th May 2012]; Available from: <http://www.4dneuroimaging.com/document-downloads/1-MAN-008-AW02_6r1.pdf>.

2. Medical S. Vicorder, instructions for use. 2007.

3. Diagnoptics. AGE reader cardiovascular assessement device instructions for use.

4. obesity WCoteo. Measureing obesity - Classification and description of anthropometric data. October 1987 [cited 2012]; Available from: <http://whqlibdoc.who.int/euro/-1993/EUR_ICP_NUT_125.pdf>.

5. Horowitz JD, Heresztyn T. An overview of plasma concentrations of asymmetric dimethylarginine {(ADMA)} in health and disease and in clinical studies: Methodological considerations. Journal of Chromatography B. 2007;851:42-50.

6. Bernini P, Bertini I, Luchinat C, Nincheri P, Staderini S, Turano P. Standard operating procedures for pre-analytical handling of blood and urine for metabolomic studies and biobanks. J Biomol NMR. 2011;49(3-4):231-43. Epub 2011/03/08.

7. Tuck MK, Chan DW, Chia D, Godwin AK, Grizzle WE, Krueger KE, et al. Standard operating procedures for serum and plasma collection: early detection research network consensus statement standard operating procedure integration working group. J Proteome Res. 2009;8(1):113-7. Epub 2008/12/17.

8. PreAnalytiX. PAXgene Blood DNA Tube

For blood collection prior to genomic DNA purification using the PAXgene Blood DNA Kit. 2010 [cited 2012 16th March]; Available from: <http://www.preanalytix.com/product-catalog/blood/dna/products/paxgene-blood-dna-tube/>.

9. P T. Improving the quality of urine strip testing: The Clinitek 50 urine chemistry analyser. Euro Clin Lab. 1997;16(20).

10. Page RC, Eke PI. Case definitions for use in population-based surveillance of periodontitis. Journal of periodontology. 2007;78(7 Suppl):1387-99. Epub 2007/08/19.

11. Field CA, Gidley MD, Preshaw PM, Jakubovics N. Investigation and quantification of key periodontal pathogens in patients with type 2 diabetes. Journal of periodontal research. 2012;47(4):470-8. Epub 2012/01/10.

12. EuroQol - a new facility for the measurement of health-related quality of life. Health Policy. 1990;16(3):199-208.
